# Yttrium-90 microsphere induced gastrointestinal tract ulceration

**DOI:** 10.1186/1477-7819-6-93

**Published:** 2008-09-02

**Authors:** Christopher D South, Marty M Meyer, Gregory Meis, Edward Y Kim, Fred B Thomas, Ali A Rikabi, Hooman Khabiri, Mark Bloomston

**Affiliations:** 1 Division of Gastroenterology, Hepatology, and Nutrition; The Ohio State University Medical Center, Columbus, Ohio, USA; 2 Department of Radiation Oncology; The Ohio State University Medical Center, Columbus, Ohio, USA; 3 Division of Interventional Radiology, The Ohio State University Medical Center, Columbus, Ohio, USA; 4 Department of Surgery, The Ohio State University Medical Center, Columbus, Ohio, USA

## Abstract

**Background:**

Radiomicrosphere therapy (RT) utilizing yttrium-90 (^90^Y) microspheres has been shown to be an effective regional treatment for primary and secondary hepatic malignancies. We sought to determine a large academic institution's experience regarding the extent and frequency of gastrointestinal complications.

**Methods:**

Between 2004 and 2007, 27 patients underwent RT for primary or secondary hepatic malignancies. Charts were subsequently reviewed to determine the incidence and severity of GI ulceration.

**Results:**

Three patients presented with gastrointestinal bleeding and underwent upper endoscopy. Review of the pretreatment angiograms showed normal vascular anatomy in one patient, sclerosed hepatic vasculature in a patient who had undergone prior chemoembolization in a second, and an aberrant left hepatic artery in a third. None had undergone prophylactic gastroduodenal artery embolization. Endoscopic findings included erythema, mucosal erosions, and large gastric ulcers. Microspheres were visible on endoscopic biopsy. In two patients, gastric ulcers were persistent at the time of repeat endoscopy 1–4 months later despite proton pump inhibitor therapy. One elderly patient who refused surgical intervention died from recurrent hemorrhage.

**Conclusion:**

Gastrointestinal ulceration is a known yet rarely reported complication of ^90^Y microsphere embolization with potentially life-threatening consequences. Once diagnosed, refractory ulcers should be considered for aggressive surgical management.

## Background

The incidence of hepatocellular carcinoma continues to increase in the United States [1,2] resulting in increased patient encounters for management decisions. Furthermore, the continued underutilization of recommended cancer screening strategies [3] results in patients diagnosed with advanced stages of cancer [4] which can include liver metastases. Several novel medical and surgical approaches are available to treat these tumors when unresectable. One such treatment strategy is radioembolotherapy also known as radiomicrosphere therapy (RT) with ^90^Y microsphere radioembolization.

This radioembolization technique consists of glass (TheraSpheres^®^, MDS Nordion Inc., Ottawa, ON) or resin (SIR-Spheres^®^, Sirtex Medical Inc., Wilmington, MA) microspheres 20–40 micrometers in size which are embedded with radioactive ^90^Y [5]. Such regional therapy takes advantage of the dual blood supply of the liver. Whereas normal liver parenchyma is supplied principally by the portal system [6], the majority of hepatic tumors derive their blood supply from the hepatic artery [7]. As such, the microspheres are selectively injected into the hepatic artery circulation and on to the tumor's microsvasculature where they embolize. As ^90^Y degrades, the microspheres emit beta-radiation (mean energy 0.93 MeV, maximum energy 2.27 MeV) to an average depth of 2.4 mm localized at the tumor site [8] so as to minimize damage to the surrounding parenchyma. The half life of ^90^Y is 64.1 hours.

While the overall complication rate of the procedure is low [9], gastric and duodenal ulceration after ^90^Y radioembolization has been described [8,10-13]. Gastrointestinal ulceration is most commonly a result of arterioarterial non-target flow of the microspheres through an aberrant hepatic arterial vasculature supplying the stomach and duodenum [12] with resultant radiation damage to the affected mucosa [8].

We sought to determine the frequency of clinically relevant gastrointestinal ulceration as a complication of ^90^Y radioembolization at our institution. Furthermore, we sought to describe each patient's clinical course in an attempt to establish common presenting signs and symptoms, as well as best treatment approaches.

## Methods

Our experience with RT began in mid-2004. Since then, we have utilized RT for primary and secondary hepatic malignancies not amenable to curative resection and/or refractory to systemic chemotherapy. We reviewed the charts of all patients undergoing RT in our early experience from 2004 through 2007. All patients underwent pretreatment celiac angiography to detect the hepatic arterial distribution of the tumor. The gastroduodenal artery was not empirically embolized as patients were to have selective right or left hepatic arterial delivery of the ^90^Y microspheres (SIR-Spheres^®^, Sirtex Medical Inc., Wilmington MA) However, if angiography demonstrated vessels at high risk for non-target flow, these were embolized prior to RT. Extrahepatic shunting was evaluated using infusion of Technetium-99 labeled macroagreggated albumin (MAA) at the precise site chosen for future RT. A catheter was placed in the right or left hepatic artery. 4 mCi of Technetium-99 labeled macroaggregated albumin were instilled via the implanted catheter. Planar images were then obtained of the lungs and abdomen to quantify the degree of extrahepatic activity i.e. shunting away from the liver lesion. Patients with less than ten percent pulmonary shunting were considered good candidates for RT using full dose of ^90^Y by dosimetry according to the manufacturer's recommendations. Those with 10–20% pulmonary shunting underwent RT with decreased ^90^Y dosing. Patients with greater than 20% percent pulmonary shunting were considered unsuitable for RT. Within four weeks, ^90^Y microspheres were infused at the exact location as the MAA study. All patients underwent immediate single photon emission computed tomography (SPECT) imaging to determine the distribution of ^90^Y. Patients were monitored for six hours and discharged the following day on a steroid taper and proton pump inhibitor.

A retrospective chart review of all patients presenting for upper endoscopy after RT utilizing ^90^Y microspheres was performed. The need for an endoscopic evaluation was determined by the treating physician. If a patient was determined to have undergone an upper endoscopy after RT their chart was reviewed further for clinical information. We specifically sought to determine presenting signs and symptoms, endoscopic findings, pathology specimen reports, and clinical outcomes. The study was approved by the cancer IRB of Ohio State University.

## Results

Twenty-seven patients underwent 33 treatments with RT for colorectal metastases (N = 15), hepatocellular carcinoma (N = 4), cholangiocarcinoma metastases (N = 2), neuroendocrine metastases (N = 2), unknown primary metastases (N = 2), prostate carcinoma metastases (N = 1), and melanoma metastases (N = 1). The median follow-up from time of RT was 6 months (mean 9.7 months; range 1–48 months). One patient was lost to follow-up after the procedure. Three patients presented with gastrointestinal ulceration.

**Figure 1:**
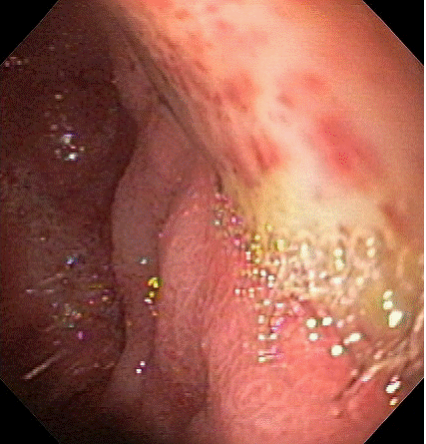
Endoscopic view of a large gastric body ulcer.

The first patient had moderately differentiated rectal adenocarcinoma metastatic to the liver. Despite aggressive cytotoxic chemotherapy his cancer progressed and the liver lesions became increasingly symptomatic with partial biliary obstruction. RT utilizing ^90^Y microspheres was determined to be the optimal treatment modality based on local expertise. Pre-RT Technetium-99 MAA showed less than 10% shunting to the lung. Pre-procedural angiography showed normal caliber vessels with the common hepatic artery trifurcating into the right hepatic artery, the left hepatic artery, and the gastroduodenal artery. The left gastric artery did not communicate with the hepatic circulation. However, the left hepatic artery trunk aberrantly arose from the common hepatic artery five millimeters proximal to the takeoff of the gastroduodenal artery and bifurcated one centimeter distal to its origin. The patient then received 1.5 GBq of ^90^Y microspheres via the right hepatic artery. Due to successful treatment with RT in the right hepatic lobe, this modality was employed for the left liver lobe lesions four months later. Normal vascular anatomy was confirmed with repeat angiography. 1.5 GBq of ^90^Y microspheres were placed in the left hepatic artery with angiographic evidence of decrease in antegrade flow. After each RT session, no evidence of extrahepatic ^90^Y deposition was seen on post-RT SPECT imaging. The patient presented 16 weeks later with a three day history of abdominal pain, nausea, and melena. Esophagogastroduodenoscopy was undertaken demonstrating erythema of the duodenal bulb and a large gastric body ulcer with a clean base (figure 1). The patient was not taking non-steroidal anti-inflammatory medications and *Helicobacter pylori *infection was not suspected although this was not specifically tested for. Biopsies were not obtained but given the lack of concomitant risk factors, complication of RT was suspected. Despite aggressive ongoing therapy with proton pump inhibitors the patient had ongoing blood loss. Gastrectomy was recommended but the patient refused further medical and surgical intervention and expired.

**Figure 2:**
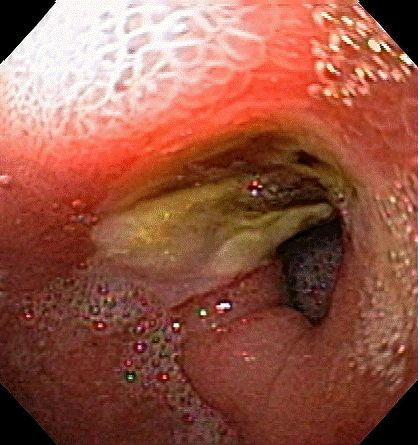
Endoscopic view of a pyloric channel ulcer.

**Figure 3:**
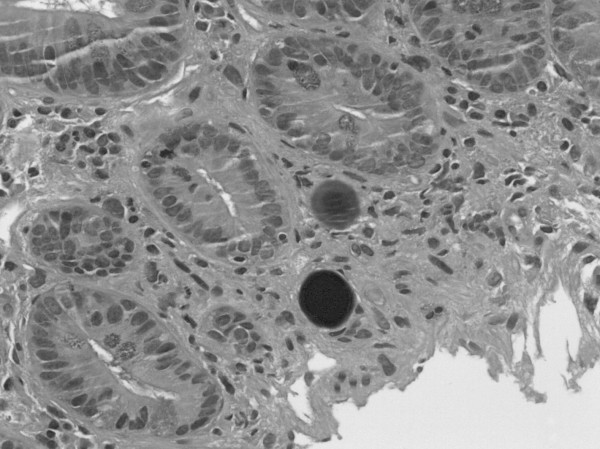
**Hematoxylin and Eosin stain of gastric biopsy specimen (400×).** Note microspheres in gastric mucosa.

The second patient had moderately differentiated adenocarcinoma of the colon originally treated by right hemicolectomy in 1994. Three years after the original diagnosis he was found to have multiple hepatic metastases and was treated with a course of 5-FU based chemotherapy. Despite aggressive medical therapy, re-staging imaging studies showed a remaining lesion in segment six. He underwent margin-negative non-anatomic resection of the solitary tumor and no other lesions were observed on intraoperative ultrasound. In 2000 he was again found to have recurrent disease in regions of margin-negative non-anatomic resection hepatic segments 4, 5, and 8. He was treated with a combination of radiofrequency ablation (RFA) and surgical resection of all disease. He again had intraoperative RFA to new lesions in the right liver in 2003. Right and left hepatic artery bland embolization was subsequently completed two months after his last operation. The patient again had cancer recurrence in his liver in 2005 and was recommended for RT. At the time of pretreatment angiography, a replaced left hepatic artery arising from the left gastric artery and several areas of narrowing in the intra-hepatic portion of the hepatic artery was seen. Prophylactic embolization of the gastroduodenal artery was completed as collateral flow to the liver supplied from this vessel was also identified angiographically. His MAA study confirmed that he had no extrahepatic shunting. One month later 1.61 GBq of ^90^Y microspheres was administered via the right hepatic artery. Post-treatment SPECT showed no extrahepatic uptake. The patient developed epigastric pain which was evaluated by upper endoscopy one month after RT. The patient was found to have a cratered gastric ulceration without bleeding at the pylorus (figure 2). No biopsies were obtained as the patient was on anticoagulant therapy. The patient was discharged on proton pump inhibitor therapy twice daily. The patient continued to have abdominal pain not relieved by acid suppressant medications and repeat endoscopy was undertaken six weeks later. Endoscopy showed a persistent non-healing ulcer in the pylorus and an additional cratered ulcer in the antrum. The patient was instructed to hold anticoagulants and biopsies of the non-healing ulcers were obtained on two separate occasions over the ensuing three months. Pathology from both specimens showed foreign material and microscopic spherules (figure 3) consistent with the patient's known history of ^90^Y therapy. The withdrawal of anti-coagulation and the continued acid suppression resulted in stabilization of the patient's hemoglobin and his symptoms resolved. The patient ultimately developed extrahepatic recurrence and expired from complications of metastatic disease.

**Figure 4:**
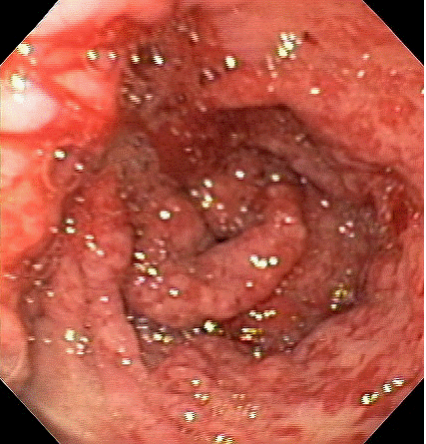
Endoscopic view of antral erosions and erythema.

The third patient had adenocarcinoma from an unknown primary metastatic to the right lobe of the liver. She subsequently underwent mesenteric angiography to evaluate her candidacy for ^90^Y RT. The patient demonstrated normal hepatic arterial anatomy including the right hepatic arterial target. There was no abnormal communication of the left gastric artery with the hepatic circulation. Technetium-99 MAA demonstrated a 5% shunt fraction to the lungs. In a second procedure, she received 1.9 GBq ^90^Y RT delivered selectively to the right hepatic artery. Stagnation of blood flow was witnessed at the conclusion of the procedure. Post-infusion scintigraphy demonstrated no radiopharmaceutical uptake outside the liver. The patient subsequently developed melena five months after RT. Upper endoscopy demonstrated friability and granularity in the duodenal bulb as well as the second portion of the duodenum and the gastric antrum (figure 4). There was a sharp demarcation of abnormal to normal mucosa in the second portion of the duodenum. Biopsies were obtained and demonstrated foreign body spherules in the gastric as well as the duodenal specimens consistent with ^90^Y therapy. *Helicobacter pylori *was not demonstrated on antral biopsies. Despite continued therapy with proton pump inhibitors, the patient continued to demonstrate gastrointestinal hemorrhage. The patient ultimately underwent empiric embolization of her gastroduodenal artery (GDA) starting at the proximal gastroepiploic artery. A combination of coils and gel foam was used to achieve successful embolization. Despite embolization of her GDA, the patient continued to have gastrointestinal hemorrhage. Consequently, she underwent repeat angiography with embolization of two small antral branches off of the left gastric artery resulting in hemostasis. However, repeat endoscopy seven months after ^90^Y radiotherapy demonstrated active bleeding from the duodenal erythema requiring epinephrine injection and argon plasma coagulation for hemostasis. Despite aggressive endoscopic therapy the patient continued to have transfusion requiring mucosal hemorrhage. Selective bland embolization of multiple left gastric artery branches was completed and her hemoglobin remained stable thereafter. The patient subsequently developed rapid progression of her cancer and expired shortly thereafter from complications of her metastatic disease. At the time of her death she did not have evidence of gastrointestinal hemorrhage.

## Discussion

In our experience, three of twenty-seven (11.1%) patients presented with endoscopically confirmed gastrointestinal ulceration/mucosal disruption. These all occurred in our first twelve cases and no changes occurred in our procedural technique during the time period studied to account for these complications. Although we have not seen any further incidents of gastrointestinal ulceration as we have gained more experience, this is possibly an underestimation as patients frequently present with non-specific abdominal complaints that may be indicative of gastrointestinal tract ulceration similar to our patients. All three of our patients presented with abdominal pain and nausea. Two of the three presented with melena. As such, clinicians should employ a low threshold for endoscopic evaluation and treatment in a patient following RT therapy with abdominal complaints or unexplained anemia.

A detailed history of non-steroidal anti-inflammatory use and history of *Helicobacter pylori *infection should be obtained prior to RT to assess risk factors for gastrointestinal ulceration. Based upon the review of our patients' records, we did not identify such risk factors, however. In patients found to be at increased risk, we recommend prolonged acid suppression and eradication of *Helicobacter pylori *if found. When corticosteroids are administered in the early post-RT period, aggressive acid suppression should be undertaken as well. Furthermore, biopsies should be obtained at the time of endoscopy to rule out an infectious etiology and to determine if foreign body spherules or radiation changes are present.

Previous experiences [8] have shown a lower (3.8%) and much higher (20%) [14] incidence of symptomatic gastroduodenal ulcerations. This lower complication rate was reported to be a result of empiric coil embolization of the gastroduodenal artery and all other collateral vessels communicating with the gastrointestinal tract at the time of angiography. In the first patient the short segment between the left hepatic artery and the GDA may have resulted in an easier retrograde reflux of microspheres into the GDA resulting in shunting of microspheres to the gastrointestinal tract. The second patient had previously received hepatic artery embolization resulting in small sclerotic hepatic artery vasculature that may have contributed to impedance of forward flow of the microspheres despite empiric GDA embolization. The anatomic variant of the left hepatic artery arising from the left gastric artery also may put the patient at risk for retrograde flow of the microspheres. No risk factor for non-target flow was identified angiographically in the third patient pre-RT. Stagnation of blood flow peri-procedurally likely contributed to retrograde flow of the microspheres through the GDA. While the routine embolization of the gastroduodenal artery was not advocated by all at the time of our study period, it has become common practice presently. Our current practice is to selectively embolize the gastroduodenal artery and any vessel at risk for shunting to the gastrointestinal tract. In addition, we periodically re-verify patency of the target vessel throughout the ^90^Y injection process as stagnation of flow may result in redirection of microspheres away from the hepatic circulation.

Furthermore, if ^90^Y microspheres are detected in biopsy specimens, medical treatment including high dose proton pump inhibitor therapy should be employed. Interventional radiologic techniques are often successful, but the optimal management strategy to treat gastrointestinal hemorrhage as a complication of RT is unknown and an early aggressive surgical approach to remove affected areas should be considered if other methods have failed.

## Conclusion

Gastrointestinal ulceration is a known and relatively common complication that is not often reported following ^90^Y microsphere embolization with potentially life-threatening consequences. Since vague upper abdominal discomfort is common after RT and often not thoroughly evaluated, the true incidence of occult ulceration is not known but occurs in at least 11% of patients despite comprehensive pre-treatment angiographic evaluation when empiric gastroduodenal artery embolization is not performed.

## Competing interests

The authors declare that they have no competing interests.

## Authors' contributions

CDS assisted in chart review and drafted the manuscript. MMM, and GM assisted in chart review and helped to draft the manuscript. EYK, AAR, and HK assisted in interpretation of radiologic procedures and revision of the manuscript. FBT participated in the design of the study and revision of the manuscript. MB conceived of the study, participated in its design and coordination, helped to draft the manuscript, and has given final approval of the version to be published. All authors read and approved the final manuscript.
